# Metabolomics and psychological features in fibromyalgia and electromagnetic sensitivity

**DOI:** 10.1038/s41598-020-76876-8

**Published:** 2020-11-24

**Authors:** Cristina Piras, Stella Conte, Monica Pibiri, Giacomo Rao, Sandro Muntoni, Vera Piera Leoni, Gabriele Finco, Luigi Atzori

**Affiliations:** 1 Department of Biomedical Sciences, University of Cagliari, Cittadella Universitaria, Monserrato, CA Italy; 2 Department of Education, Psychology and Philosophy, University of Cagliari, Cagliari, Italy; 3 National Institute for Occupational Accident Insurance (INAIL), Rome, Italy; 4 Department of Medical Sciences, University of Cagliari, Cagliari, Italy

**Keywords:** Biomarkers, Diseases

## Abstract

Fibromyalgia (FM) as Fibromyalgia and Electromagnetic Sensitivity (IEI-EMF) are a chronic and systemic syndrome. The main symptom is represented by strong and widespread pain in the musculoskeletal system. The exact causes that lead to the development of FM and IEI-EMF are still unknown. Interestingly, the proximity to electrical and electromagnetic devices seems to trigger and/or amplify the symptoms. We investigated the blood plasma metabolome in IEI-EMF and healthy subjects using ^1^H NMR spectroscopy coupled with multivariate statistical analysis. All the individuals were subjected to tests for the evaluation of psychological and physical features. No significant differences between IEI-EMF and controls relative to personality aspects, Locus of Control, and anxiety were found. Multivariate statistical analysis on the metabolites identified by NMR analysis allowed the identification of a distinct metabolic profile between IEI-EMF and healthy subjects. IEI-EMF were characterized by higher levels of glycine and pyroglutamate, and lower levels of 2-hydroxyisocaproate, choline, glutamine, and isoleucine compared to healthy subjects. These metabolites are involved in several metabolic pathways mainly related to oxidative stress defense, pain mechanisms, and muscle metabolism. The results here obtained highlight possible physiopathological mechanisms in IEI-EMF patients to be better defined.

## Introduction

Fibromyalgia (FM) is a complex multiorgan system disease with unknown etiology^1^. According to 2016 revisions to the fibromyalgia diagnostic criteria, fibromyalgia may now be diagnosed in adults when all of the following criteria are met: (1) generalized pain, symptoms have been present at a similar level for at least 3 months, (2) Widespread pain index (WPI) ≥ 7 and symptom severity scale (SSS) score ≥ 5 OR WPI of 4–6 and SSS score ≥ 9, (3) a diagnosis of fibromyalgia is valid irrespective of other diagnoses^2^. Thus, FM patients show a significant reduction in their quality of life and are frequently absent from their workplace. Several attempts have been unsuccessfully made to identify reliable, sensitive, and measurable biomarker, so that, currently, the diagnosis is mainly clinical^1^. However, some studies have shown a condition of oxidative stress, quantifiable by measurement of reactive oxygen species amount, as a relevant element in the pathogenesis of FM^3–5^.

Due to the increased electrosmog exposure, concerns about the likely harmful effects of the extremely low-frequency magnetic, and both low and high electrical fields have increased over the last decades. Accordingly, some people reported severe symptoms in the proximity of electrical devices operating at various frequency ranges. This phenomenon is called idiopathic environmental intolerance attributed to electromagnetic field (EMF) which is associated with non-specific physical symptoms (NSPS) appearing when an electromagnetic field source is present and perceived by an individual^6^. This condition can have major implications with general health status decline, increased distress, and health service use and impairments in occupational and social functioning^7–9^. Like FM, IEI-EMF is a highly disabling pathology characterized by dermatological, neurological, vegetative and cognitive symptoms. Among these, the prevailing are allergies, food and drugs intolerances, stress, backache, abnormal fatigue, muscle tension, skin dryness, joint pain, headache, photosensitivity and sleep disorder^10^, dizziness, difficulties in concentration, memory problems, anxiety, respiratory problems (e.g. difficulties of breathing), gastrointestinal symptoms, eye and vision symptoms (e.g. double vision and blurred vision), palpitations and so on^11^.

A survey performed in 5 European countries (France, Germany, Italy, Portugal, and Spain) estimated 4.7% of the prevalence of fibromyalgia in the general population^12^. Health consequences can be serious for long-ill patients who are often forced to leave work and move from their homes.

Some studies focused on the probable causal link between health worsening and exposure to EMF. The trigger of health problems may be the continuous exposure to different electrical devices and appliances such as computers, general office equipment, fluorescent lights, household appliance, television etc. These problems can worsen with time as indicated by the relatively poor prognosis^11^. The dangerous effects related to EMF are well described in the literature and sensitive subjects could be likely affected by low and high levels of EMF exposure. Nevertheless, there is the assumption that only the acute exposure to EMF is dangerous to human health, whereas the chronic one can be neglected. Many cases of childhood leukemia and tumors in adults due to occupational and residential exposure to electric fields or EMF have been reported^13–19^. Moreover, it has been associated with increased incidence of spontaneous abortion^20^ and neurodegenerative diseases^21^, such as Parkinson and Alzheimer disease^20^ and lateral amyotrophic sclerosis^22^. Noteworthy, the increased risk was found at magnetic field levels comparable with the ones present in a residential situation (0.2–5.0 Mt)^23^. Furthermore, Gennaro et al.^24^ reported neoplastic and pre-neoplastic effects in rodents irradiated with EMF at levels corresponding to those associated to human exposure, considering the different exposure conditions and the different lifespan between humans and rodents (to 0.3 µτ in residential exposure to power lines)^25^.

Some researchers interpret NSPS related to IEI-EMF as the outcome of anxiety, depression, somatization, symptoms of exhaustion, and stress^26^. Other defined NSPS symptoms to be related to certain personality traits and cognitive-emotional factors (e.g. somatizations tendency, somatosensory amplification, elevated risk perception, worries about the possibility of harmful features of modern life called modern health worries)^9,27^. Thus, these symptoms are considered of psychogenic origin^28^ or associated with the so-called nocebo phenomenon^6,7^.

In the present study, we conducted a plasma metabolomics characterization of patients with IEI-EMF and FM compared to a control group. Metabolomics is a powerful analytical tool used for the identification of low molecular weight molecules, able to capture disease‐specific metabolic signatures as possible biomarkers. In the last 10 years, metabolomics has been applied widely and successfully in various fields of medicine for the study and discrimination of various pathologies such as cardiovascular^29,30^ and neurodegenerative and psychiatric diseases^31,32^, as well as cancer^33^. To date, the pathogenesis of FM and specifically of IEI-EMF is completely unknown. In this study, we used metabolomics analysis as a tool to better understand the pathogenetic mechanisms that support FM and identify disease-specific biomarkers^34^. Furthermore, to verify the psychogenic origin of IEI-EMF, patients and control groups were tested for anxiety, locus of control, and personality.

## Results

### Multivariate analysis of variance: psychosocial descriptors

In accordance with the BFQ theory, the differences in personality, anxiety in state, and in trait locus of control were evaluated with multivariate analysis of variance (MANOVA and ANOVA) to ascertain differences between IEI-EMF subjects and controls. MANOVA showed no significant differences between the two groups (F = 0.91; df = 7/49; p = 0.51). ANOVA results with factor groups (IEI-EMF subjects and controls) and with dependent variable “Energy”, “Friendship”, “Conscientiousness”, “Emotional stability”, “Openness”, STAI-Y (State), STAI-Y (Trait), and Locus of Control showed no significant differences between IEI-EMF subjects and controls (p > 0.05) (Table 1).

**Table 1:** Psychological variables and FIQ (Fibromyalgia Impact Questionnaire) (means and standard deviations).

	IEI-EMF	Controls	F (df)	p
Energy-Extraversion	78.71 (± 11.28)	76.43 (± 8.93)	0.99 (1/49)	0.32
Friendship	84.90 (± 11.59)	86.30 (± 11.61)	0.03 (1/49)	0.85
Conscientiousness	81.10 (± 10.19)	81.85 (± 9.87)	0.69 (1/49)	0.41
Emotional Stability	67.72 (± 18.01)	73.28 (± 14.25)	0.48 (1/49)	0.49
Opennes	85.48 (± 14.26)	91.33 (± 10.15)	2.11 (1/49)	0.15
Stay-Y State	34.85 (± 14.26)	32.33 (± 11.04)	0.95 (1/49)	0.33
Stay-Y Traits	40.33 (± 12.33)	37.61 (± 11.04)	0.47 (1/49)	0.49
Locus of Control	17.66 (± 13.55)	24.14 (± 17.30)	0.89 (1/49)	0.35
FIQ	66.23 (± 12.3)	–	–	–

### Metabolomics

#### Multivariate statistical analysis

**Figure 1:**
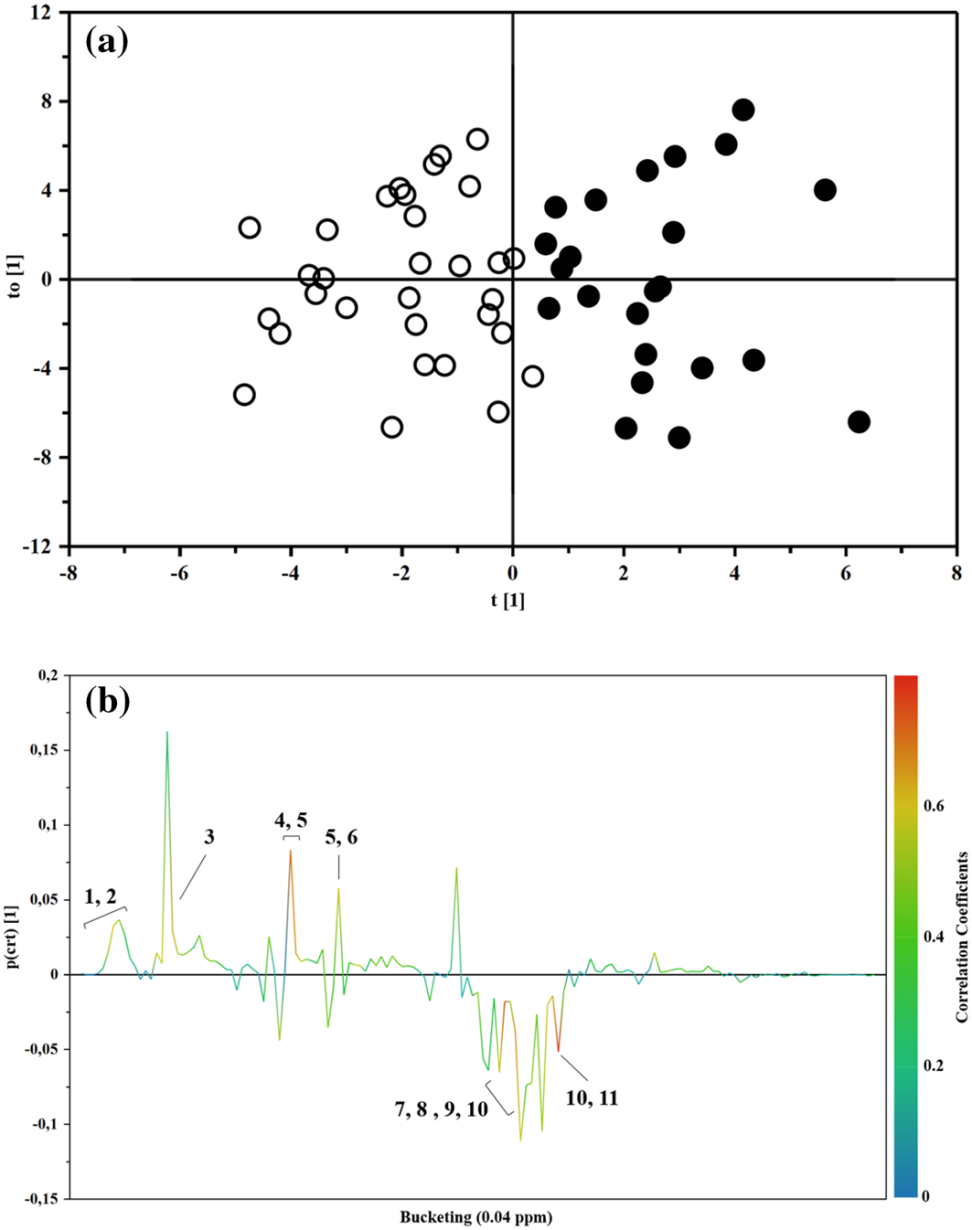
(**a**) OPLS-DA scores plot of ^1^H-NMR spectra of plasma samples: Controls (full circle), IEI-EMF subjects (open circle). (**b**) Color-coded coefficient loadings plot of metabolomics profile between Controls and IEI-EMF subjects. Peaks: **1 and 2**, 2-hydroxyisocaproate and Isoleucine; **3**, Lactate; **4 and 5**, Homoserine and Glutamine; **5 and 6**, Glutamine and Pyroglutamate; **7, 8, 9 and 10**, Glutamine, Glycine, Myo-inositol and Choline; **10 and 11**, Serine and Choline.

^1^H-NMR spectroscopy coupled with multivariate data analysis was applied to investigate the metabolomics profile of plasma samples for both IEI-EMF subjects and controls. ^1^H-NMR spectra of plasma samples from controls and IEI-EMF subjects are shown in Supplementary Fig. S1. Each ^1^H-NMR spectrum can be divided into two main spectral zones: the region between 0.5–5.5 ppm characterized by a large number of partly overlapping peaks due to the aliphatic groups of free amino acids, organic acids and sugars, and the region between 6.8–8.5 ppm characterized by the signals from the aromatic metabolites. The whole ^1^H-NMR dataset was subjected to multivariate statistical analysis. PCA analysis (data not shown) was performed to evaluate the homogeneity of the samples in each group (IEI-EMF and controls) and identify potential outliers (outside the 95% confidence limit). Both IEI-EMF and control groups resulted in particularly consistent results and showed no outliers. To remove potential information not related to the disease of interest and highlight possible metabolic differences between IEI-EMF subjects and controls, and OPLS-DA analysis was subsequently conducted on the same dataset. OPLS-DA scores plot (Fig. 1a) showed good separation between IEI-EMF subjects and controls indicating differences in the metabolomics profile between the two groups.

The OPLS-DA model was established with one predictive and two orthogonal components and showed good values of R2X, R2Y, and Q2 (Table 2). The validity of the OPLS-DA model was evaluated through a permutation test (Supplementary Fig. S2) using 500 times. The test results are reported in Table 2 and indicate the statistical validity of the OPLS-DA model. The S-line plot was used to identify the potential metabolites that contributed to the plasma metabolome modification in IEI-EMF subjects compared to controls (Fig. 1b). A *p(corr)* > 0.6 was selected as a significance level.

**Table 2:** Statistical parameters for OPLS-DA model.

OPLS-DA model using the whole ^1^H-NMR metabolomics profile						
	Components^a^	R2Xcum^b^	R2Ycum^c^	Q2cum^d^	R2 intercept	Q2 intercept
IEI-EMF *versus* controls	1P + 2O	0.639	0.699	0.557	0.239	− 0.439

^a^The number of Predictive and Orthogonal components used to create the statistical models.

^b,c^ R2X and R2Y indicated the cumulative explained fraction of the variation of the X block and Y block for the extracted components.

^d^ Q_2_cum values indicated cumulative predicted fraction of the variation of the Y block for the extracted components.

*R2 and Q2 intercept values are indicative of a valid model.

#### Significant metabolites identification

The metabolic profile of IEI-EMF was characterized by changes in different spectral regions with overlapped signals due to different metabolites such as 2-hydroxyisocaproate, choline, glucose, glutamine, glycine, homoserine, isoleucine, lactate, myo-inositol, pyroglutamate, serine, and taurine.

**Figure 2:**
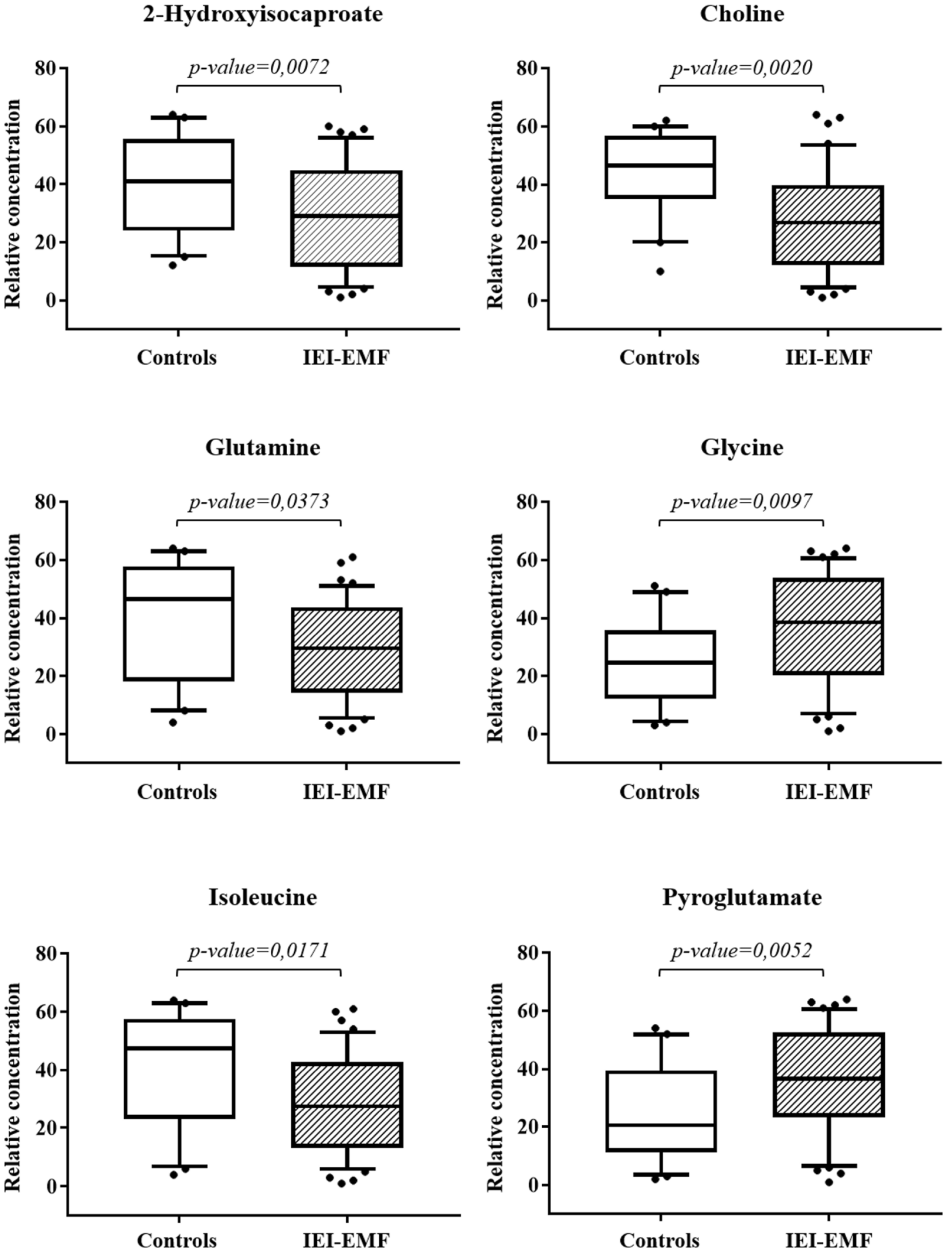
Box-and-whisker plots showing progressive changes of the metabolites concentration on Controls and IEI-EMF plasma samples. Statistical significance was determined using the Mann–Whitney U test and a p-value < 0.05 was considered statistically significant. The Holm-Bonferroni adjustment was applied.

**Figure 3:**
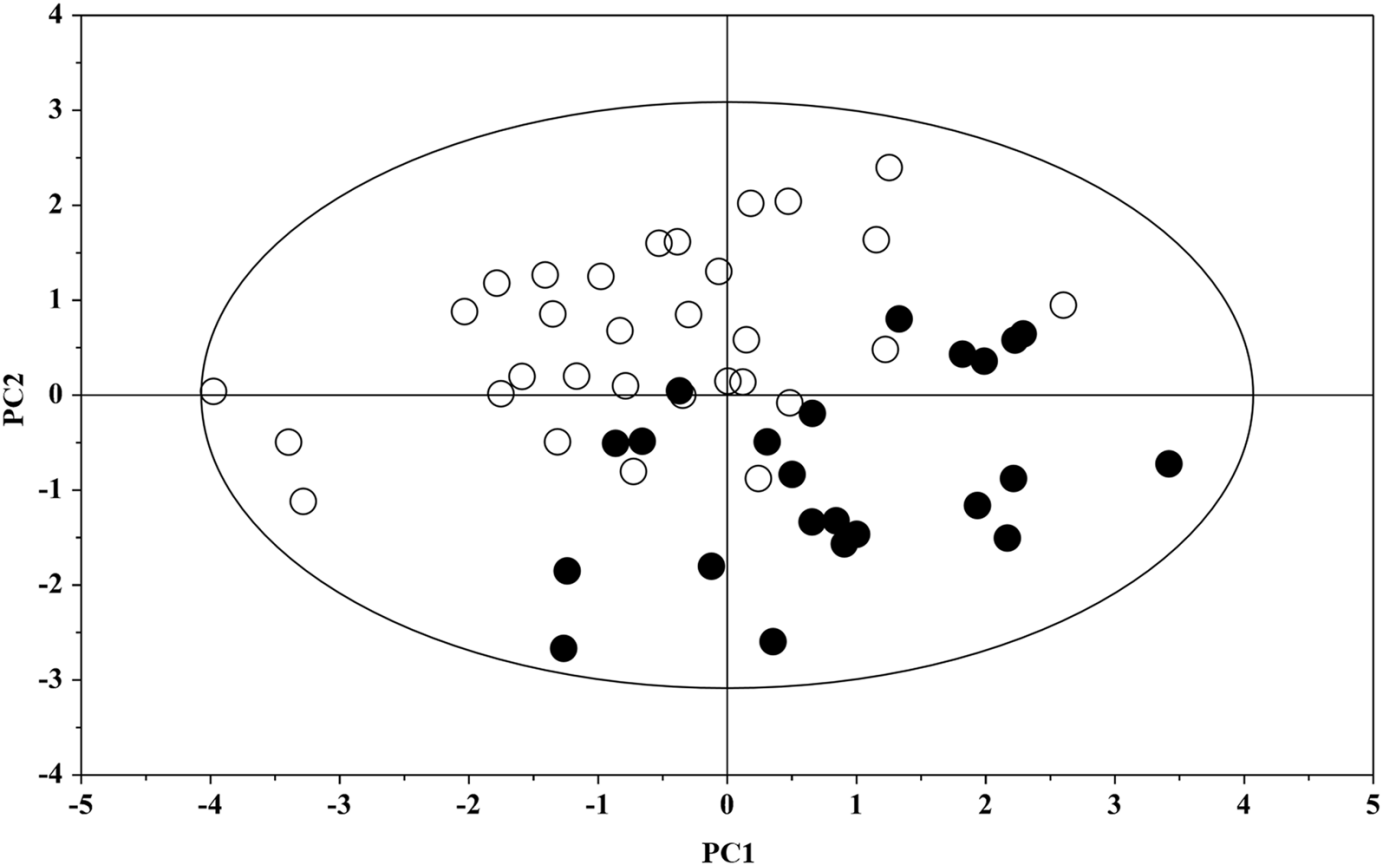
PCA scores plot built with only identified significant metabolites: Controls (full circle), IEI-EMF subject (open circle).

To evaluate the actual importance of the individual metabolites, the spectral regions highlighted in the S-line plot were quantified by Chenomx NMR Suite 7.1. and subjected to Mann–Whitney U test to identify significant variations of their concentration in the two groups. The results of the univariate statistical analysis showed that only 2-hydroxyisocaproate, choline, glutamine, glycine, isoleucine, and pyroglutamate changed significantly in IEI-EMF subjects compared to controls (with p-value < 0.05). The relative concentrations of these metabolites in the two groups were compared using box-and-whisker plots. As shown in Fig. 2, IEI-EMF subjects were characterized by a higher level of glycine and pyroglutamate, and lower levels of 2-hydroxyisocaproate, choline, glutamine, and isoleucine compared to controls. Then, a new PCA model was constructed using only the identified significant metabolites and the result of the analysis is shown in Fig. 3. Figure 3 shows the projection of the samples on the plane formed by the first two PCs that explain 62.2% of the total variance and good separation between IEI-EMF and controls was observed, indicating how the identified metabolites play an important role in the separation of the two groups.

**Figure 4:**
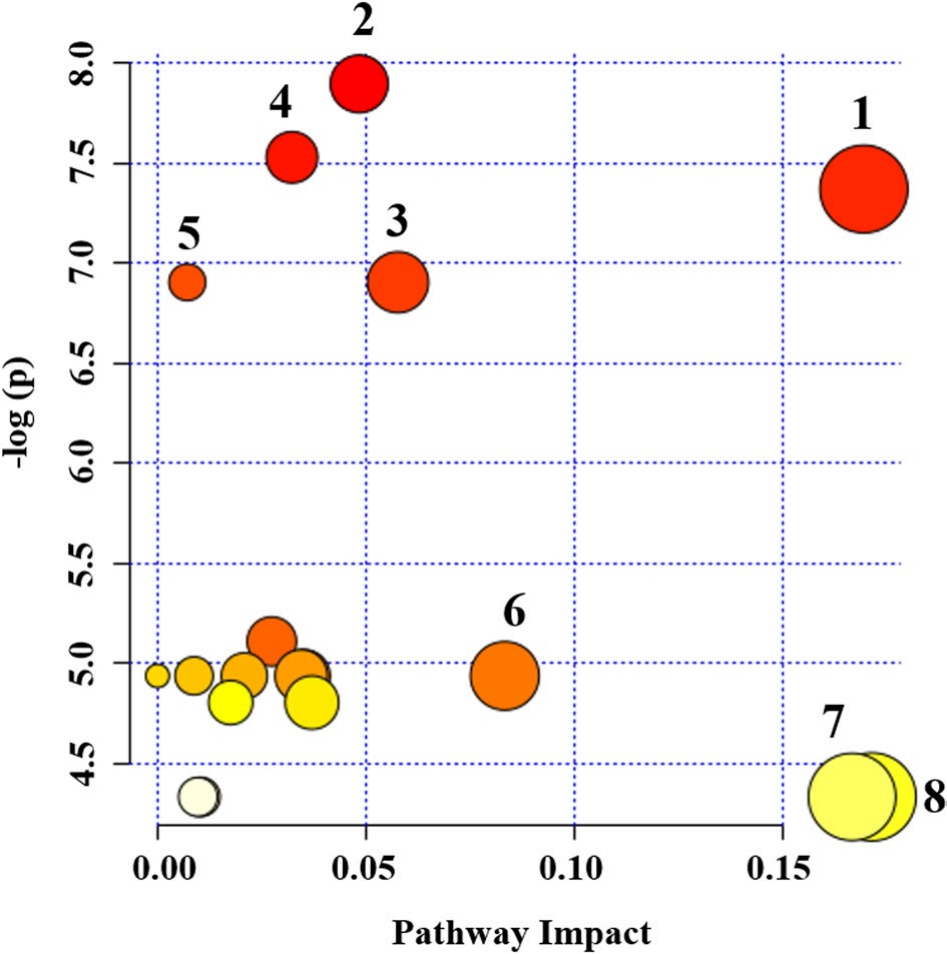
Summary of pathway analysis of IEI-EMF group compared to Controls. Plot was obtained by using MetaboAnalyst 4.0. “X axis” represents the impact of the identified metabolites on the indicated pathway. “Y axis” indicates the extent to which the designated pathway is enriched in the identified metabolites. Circle colors indicate pathway enrichment significance. Circle size indicates pathway impact. **1**, Glycine, serine and Threonine metabolism; **2**, Aminoacyl-tRNA biosynthesis; **3**, Nitrogen metabolism; **4**, Glutathione metabolism; **5**, Purine metabolism; **6**, Cyanoaminoacid metabolism; **7**, Alanine, aspartate and glutamate metabolism and **8**, d-glutamine and d-glutamate metabolism.

The pathways analysis, performed using all the significantly different metabolites resulting from the multivariate analysis, underlined Aminoacyl-tRNA biosynthesis, Glutathione metabolism, Purine metabolism, Nitrogen metabolism, Glycine, Serine and Threonine metabolism, Cyanoaminoacid metabolism, D-Glutamine and D-glutamate metabolism and Alanine, aspartate and glutamate metabolism as the most important networks (Fig. 4).

## Discussion

The IEI-EMF is a disorder potentially due to exposure to magnetic fields produced by electrical devices of common daily use. As the pathogenesis of IEI-EMF is currently controversial and little is known, to better characterize the mechanisms associated with its onset here we investigated the blood plasma metabolome of IEI-EMF patients and healthy subjects using ^1^H-NMR spectroscopy coupled with multivariate statistical analysis. To better understand the metabolomics results, it was of fundamental importance to completely exclude that the symptomatology described by IEI-AMF patients was dependent on psychological disorders. Thus, patient psychological characteristics were evaluated through the administration of three tests, named *Big Five Questionnaire*, *Locus of Control Test* and *Stai-Y test*. Data obtained, showed no significant psychological differences between IEI-EMF patients and controls. It is important to emphasize that IEI-EMF patients did not take specific pharmacological treatments for the reduction of pain and other comorbidities that accompany their daily lives. So, this feature likely has led them to have a better compensatory cognitive strategy to control anxiety and, by using some precautions (such as avoiding long exposures to EMF and using specific shielding clothing), a better adaptation to the environment. Opposite, and with the caveat that are preliminary data, the IEI-EMF patients showed alterations of the metabolomics profile markedly distinguished from the controls, suggesting that the symptomatology of IEI-EMF patients can be of pathological and not psychological nature. The separation between the two groups (IEI-EMF subjects and controls) was mainly due to a particular set of metabolites. These metabolites are involved in several pathways, such as glutathione metabolism, purine metabolism, nitrogen metabolism, and, in general, the amino acid metabolism. In particular, the IEI-EMF subjects showed significantly higher levels of glycine and pyroglutamate and lower levels of 2-hydroxyisocaproate, choline, glutamine, and isoleucine compared to controls. The pain is a predominant symptom in IEI-EMF subjects, and in general in those affected by fibromyalgia. Indeed, in 95% of cases, constant and diffuse pain is mainly localized in the limbs and torso^35,36^. Some Magnetic Resonance Imaging studies conducted to evaluate brain metabolism in patients with fibromyalgia were able to highlight the activation of the same brain areas triggered by painful stimuli (called "pain matrix") through the analysis of specific molecules such as N acetylaspartate, creatine, choline, lactate, myoinositol, glutamine and glutamate^37–39^.

Then, the primary hypothesis on the pathogenesis of fibromyalgia highlights the role of the central nervous system in the amplification of pain perception and the development of other co-morbid symptoms, such as sleep-related problems, fatigue, emotional distress, and cognitive difficulties.

The metabolomics analysis here reported, have shown lower choline levels in the plasma of IEI-EMF subjects compared to controls. Choline is a marker of phospholipid metabolism and participates in cellular membrane turnover and osmotic regulation in glial cells. Previously, Fayed et al. have found^40,41^ a lower concentration of choline in hippocampal and posterior cingulate cortex areas in FM patients compared to healthy subjects. Furthermore, some authors reported that the change in choline levels provides a sensitive indication of altered brain metabolic activity^42,43^. The decrease in choline concentration is associated with osmolar changes in the brain, in particular, it seems to work as a compensatory response to the increased intracellular osmolarity caused by the accumulation of glutamine in astrocytes. Based on this, the decline in choline concentration observed in our IEI-EMF patients could be explained as a marker of altered cerebral activity which could be responsible for the cognitive symptoms (such as confusion, forgetfulness, anxiety) appearing in these subjects in presence of EMF source^6^. Compared to controls, IEI-EMF patients were also found to have higher levels of glycine. Glycine is an inhibitory transmitter in the spinal cord and a positive modulator of the *N*-methyl-d-aspartate receptor (NMDAr), which is thought to be involved in nervous system reorganization and chronic pain in fibromyalgia patients^44,45^. The NMDAr shows increased activity in fibromyalgia and several studies have analyzed receptor modulation as a target for therapeutic intervention^46^. When is in its inactive state, the NMDAr is bound to extracellular magnesium and zinc and prevents the flow of cations through the synaptic channel. Receptor activation occurs after binding to glutamate and glycine amino acids at different receptor sites. It results in calcium influx, which triggers neuronal excitation and intracellular signaling cascades involved in synaptic plasticity processes^46–48^. The malfunctioning of the spinal inhibitory input on the central pain circuits shows a crucial role in the facilitation and maintenance of chronic pain. Some research groups have shown that glycine-mediated synaptic inhibitory neurotransmission in the spinal cord dorsal horn suppresses pain. Furthermore, inhibition of glycine reuptake and positive allosteric modulation of the glycine receptor has been shown to increase spinal glycinergic tone and improve pain behaviors in various rodent models of acute, inflammatory, and neuropathic pain^47,49,50^. The metabolomics analysis of IEI-EMF patients compared to controls has also shown low levels of isoleucine, glutamine, and end-products of leucine metabolism. Isoleucine and leucine, are branched-chain amino acids (BCAAs) and are involved in stress, energy, and muscle metabolism. Resting muscle metabolizes BCAAs and transamination amino acids to produce ATP through the tricarboxylic acid cycle (Supplementary Fig. S3).

Several authors^51–53^ have suggested that an alteration in energy metabolism may be present in some of the muscle fibers of fibromyalgia patients and that this may determine many of their symptoms, including generalized pain. It has been hypothesized that a reduction in plasma and urine concentrations of BCAAs, such as isoleucine, leucine e valine could be associated with potential muscle depletion. Accordingly, many studies have shown that BCAAs supplementation may decrease muscle catabolism, reducing central fatigue, through increased competition for the cerebral uptake mechanism of tryptophan^54–56^. The decreased levels of BCCAs may affect the body glutamate-glutamine pool leaving the tissues more vulnerable to oxidative stress. Indeed, compared to controls, the IEI-EMF patients showed a lower concentration of glutamine. Glutamine has numerous functions including glutathione production^57^, muscle protein synthesis^58^, gut health, maintenance of acid–base balance in the kidney^59^, and removal of toxic ammonia from the tissues. Glutathione is one of the most important antioxidant molecules, and a limited supply of glutamine in the body could cause low levels of glutathione determining oxidative stress-associated damage. Thus, in a study conducted on patients suffering from chronic fatigue^60^, glutamine supplementation has been suggested as a mean to support glutathione pathway deregulation. An alteration of the glutathione pathway is consistent with the increased concentration of glycine and pyroglutamate observed in IEI-EMF patients. The dipeptide γ-glutamylcysteine, formed in the first step of glutathione synthesis, can be the substrate for two different enzymes: γ-glutamylcyclotransferase (which produces pyroglutamate) and GSH-synthetase (which uses glycine to produce glutathione)^61^. Several studies have shown that oxidative stress due, for instance, to metal and / or drug toxicity, may lead to glutathione depletion with consequent accumulation of pyroglutamate in the urine^62^, considered a specific marker for glutathione depletion^63^. Finally, in IEI-EMF patients were found lower 2-hydroxyisocaproate levels compared to controls. The 2-hydroxyisocaproate derives from leucine metabolism by transamination in human tissues, such as muscle and connective tissue. It can be considered as an anti-catabolic substance and some studies have shown a decreased muscle protein degradation associated with its intravenous infusion^64,65^. Moreover, it is a potent inhibitor of branched-chain α-ketoacid dehydrogenase kinase, which may lead to increased catabolism of BCAAs. The catabolism in the muscle is associated with the breakdown of muscle proteins and delayed-onset muscle soreness. Several studies indicate that the administration of BCAAs, in particular leucine, and their transaminated metabolites, such as 2-hydroxyisocaproate, can effectively relieve muscle pain symptoms and protect muscle from catabolism. In this view, the lower levels of 2-hydroxyisocaproate observed in our IEI-EMF patients could be responsible of the muscle weakness, which manifests as abnormal fatigue and muscle tension.

## Conclusion

To the best of our knowledge, this is the first study dealing with FM and electromagnetic sensitivity together (IEI-EMF). We could not find any study focusing on the relationship between IEI-EMF and metabolomics. This aspect is the main element of the novelty of this current study**.**

The results obtained from our metabolomics study demonstrate how IEI-EMF patients are characterized by a significantly different metabolomics profile compared to control subjects. The most significantly altered pathways appear the ones correlated to oxidative stress defense and pain control. The results show no significant differences between IEI-EMF patients and controls for personality aspects, Locus of Control, and anxiety. The cohort of IEI-EMF patients studied is not fully representative of the FM population, as the patients were "highly motivated". The almost total overlap between healthy controls and IEI-EMF patients for psychological characteristics may be due to increased resilience, hope, and optimism in subjects with chronic invalidating, but non-fatal disease. They tend to hope that a better solution can be found for their pathology. Although preliminary and with some limits, our data, indicate the presence of metabolic changes suggesting a possible physiopathological mechanism. A limitation of our study is the population under investigation including only fibromyalgic patients with electromagnetic sensitivity. We could not find patients with IEI-EMF without FM. We started from the general assumption that, while all the IEI-EMF are also FM, not all FM are IEI-EMF as well. We agree that further studies to better define the role of FM vs IEI-EMF are needed. So, in our study, it is not possible to differentiate effects which are FM- or IEI-EMF-related. Some of the differences observed may not be associated with IEI-EMF but to FM too. In a future study, another group of patients with the only FM would be evaluated. Validation of the present results may lead to new biomarkers discovery and therapeutic approaches.

## Materials and methods

### Characteristics of the study population

The study was carried out on 54 subjects: 31 affected by FM and electromagnetic sensitivity, IEI-EMF (30 females and 1 male), and 23 controls (21 females and 2 males). The demographic characteristics of the population under study are shown in Table 3.

**Table 3:** Demographic characteristics of the population (means and standard deviations).

	IEI-EMF	Controls
Age	47.46 (± 11.28)	45.86 (± 10.43)
Schoolarity	13.90 (± 4.23)	15.23 (± 3.71)
**Main food intolerance of IEI-EMF (number of patients)**		
Foods containing nickel	6	–
Foods containing lactose	14	4
Foods containing gluten	15	–
Fruits	8	–
Solanaceous	11	–
**The most frequent symptoms of IEI-EMF (number of patients)**		
Burning or pain of hands	16	–
Burning or pain of legs	12	–
Burning or pain of shoulder	14	–
Burning or pain of foot	14	–
Laryngitis	10	–
Pharyngitis	13	–
Tinnitus	13	–
Dizziness	14	–
Photosensitivity	15	–
Difficulty in breathing	12	–
Tachycardia	11	–
Chills	15	–
Digestive slowness	22	–
Abdominal swelling	23	–
Irritable bowel	13	–
Heartburn	16	–
Bloating	14	–
Cystitis	10	–
**Electromagnetic exposure at home and work**		
Main road	30	23
Nuclear site (less than 10 km)	13	3
Steel Industries	17	10
Mobile towers (less than 1 km)	31	14
Power lines	26	11
Dumps (less than 5 km)	19	12
Industries of Chemicals (less than 5 km)	21	8
Airport (less than 5 km)	14	7
Refinery (less than 5 km)	17	5

The participants filled in a questionnaire about their dietary habits to identify intolerance (or food avoidance). Most IEI-EMF subjects (73%) showed intolerances particularly for milk and derivatives, wheat, and *Solanaceae* (Table 3).

The patients needed a previous diagnosis of FM to take part in this current study. Furthermore, according to the American College of Rheumatology (ACR)^66,67^ they were required to fill in a questionnaire about their symptomatology to ascertain the presence of FM. The questionnaire for symptoms was scored as 0 = no problem; 1 = slight problem (intermittent); 2 = moderate: considerable problem (often detected); 3 = severe problem (pervasive, continuous, affecting the quality of life).

Furthermore, the major inclusion criteria used to identify individuals with IEI-EMF were^68^: (1) attribution of NSPS to either various or specific source of EMF; (2) self-reported IEI-EMF; (3) experience of symptoms during or soon (from 20 min to 24 h) after the presence or use of an EMF exposure source; (4) high score on a symptom scale (corresponding to pervasive or considerable) in the presence of a EMF exposure source; (5) limitation in the daily functioning of the individual due to the EMF-related health effects. The most frequent symptoms are reported in Table 3.

All participants were asked to complete a preliminary questionnaire focused on personal characteristics as well as their personal medical and psychological history. Furthermore, they were all asked to fill in a questionnaire to assess their electromagnetic exposure at home and work and another questionnaire to assess their chemical exposure at home and work currently or in the past (Table 3). The details of the questionnaire about electromagnetic exposure and chemical exposure are reported in Supporting Information.

The analysis of the questionnaires allowed us to classify the triggering events into five groups approximately: (1) chemical exposure; (2) electromagnetic exposure; (3) biological exposure (viruses, bacteria, fungi, mold, etc.); (4) high fever; (5) psychological trauma. Many patients have undergone several convergent triggering events in the same year. Conversely, subjects included in the healthy group did not present physical pathologies.

No participants took drugs 30 days before blood sampling. Someone consumed supplements and cannabinoids. The exclusion criteria for both patients and controls were: the presence of systemic diseases such as hypo- or hyperthyroidism; rheumatoid arthritis, vasculitis, diabetes mellitus, heart disorders, history of acute or chronic infections, cerebrovascular diseases, alcohol abuse, depressive disorders, psychiatric pathologies and abnormalities in routinary biochemical analyses of blood and urine.

The institutional ethics committee (University of Cagliari, Italy) approved the study and written informed consent was obtained from all participating subjects and that was conducted in accordance with the Declaration of Helsinki.

### Psychological analysis

#### Evaluation of psychological characteristics

All subjects were administered three tests to assess psychological characteristics: Big Five Questionnaire^69^, Locus of Control Test^70^ and Stai-Y test^71^. All tests are self-report questionnaires and that can be administered in individual format. The details of the tests are reported in Supporting Information.

#### Multivariate analysis of variance

A MANOVA was performed to ascertain differences between IEI-EMF subject and controls in personality according to Big Five Questionnaire theory (BFQ), State–Trait Anxiety Inventory (STAI) and Locus of Control. Factors analyzed were: groups (2 levels: patients and controls) and dependent variables: 5 for BFQ (Energy, Friendship, Conscientiousness, Emotion Stability, and Openness); 2 for Stay-Y (Stai-Y Trait and Stai – Y State) and Locus of Control.

After MANOVA, eight 1-way ANOVAs were performed to test differences between IEI-EMF subjects and controls for each dependent variable^72^.

### ^1^H-NMR analysis

#### Sample collection and preparation for ^1^H-NMR experiments

Blood samples were collected in heparinized tubes, immediately centrifuged at 4000 rpm for 15 min, and about 800 μL stored at − 80 °C until metabolomics analysis. The extraction of water-soluble metabolites from plasma samples was performed based on the Folch, Lees, and Sloane-Stanley procedure^73^ and has been already described in previous papers published^31^. 400 μL of plasma were dissolved in 1.2 mL of a chloroform/methanol mixture (1:1, v/v) and 175 µL of H_2_O. The solution was centrifuged at 4500 rpm and 4 °C for 30 min and ∼1 mL of hydrophilic phase, containing the low molecular weight water-soluble components, was separated from the lipophilic one, dried using a speed vacuum concentrator (Eppendorf, Hamburg, Germany) and then stored at − 80 °C. Dried hydrophilic plasma extracts were re-dissolved in 690 μL of potassium phosphate buffer in D_2_O (100 mM, pH 7.4) and 10 μL of TSP (sodium 3-trimethylsilyl-propionate-2,2,3,3,-d4) as chemical shift reference (δ 0.0) (98 atom % D, Sigma-Aldrich, Milan). An aliquot of 650 μL was analyzed by ^1^H-NMR.

### ^1^H-NMR spectroscopic analysis

^1^H-NMR measurements of plasma samples were carried out using a Varian UNITY INOVA 500 spectrometer operating at 499.839 MHz for proton and equipped with a 5 mm double resonance probe (Agilent Technologies, CA, USA). ^1^H-NMR spectra were acquired at 300 K with a spectral width of 6000 Hz, a 90° pulse, an acquisition time of 2 s, a relaxation delay of 2 s. For each sample, 256 free induction decays were collected into 64 K data points^74^. The residual water signal was suppressed by applying a presaturation technique with low power radiofrequency irradiation for 2 s.

After Fourier transformation with 0.3 Hz line broadening and a zero-filling to 64 K, ^1^H-NMR spectra were manually phased and baseline corrected using ACDLab Processor Academic Edition (Advanced Chemistry Development, 12.01, 2010). Spectral chemical shift referencing on the TSP CH_3_ signal at 0.00 ppm was performed on all spectra. Metabolites were identified based on literature information and by using a dedicated library, such as the Human Metabolome Database (HMDB, https://www.hmdb.ca) and the 500 MHz library from Chenomx NMR suite 7.1 (Chenomx Inc., Edmonton, Alberta, Canada)^75^. Chenomx NMR Suite is an integrated set of tools for identifying and quantifying metabolites in NMR spectra. It is equipped with reference libraries that contain numerous pH-sensitive compound models that are identical to the spectra of pure compounds obtained under similar experimental conditions. Essentially, a Lorentzian peak shape model of each reference compound is generated from the database information and superimposed upon the actual spectrum. The linear combination of all modeled metabolites gives rise to the total spectral fit, which can be evaluated with a summation line.

### NMR data preprocessing and multivariate statistical analysis

The ACD Labs intelligent bucketing method was used for spectral integration between 0.80 and 8.50 ppm^74^. A 0.04 ppm bucket width was defined with an allowed 50% looseness, resulting in buckets that ranged between 0.02 and 0.06 ppm in width. The degree of looseness allows the bucket width to vary over a particular value from the set bucket value. The intelligent bucket method contains an algorithm, which identifies local minima in the spectra and sets the buckets accordingly. In this manner, a peak is integrated into one bucket, although it may be differently shifted in the spectra because of the pH effect, for instance. The spectral region between 4.70 and 5.20 ppm was excluded from the analysis to remove the effect of variations in the presaturation of the residual water resonance. The spectral data set was normalized to the total area to minimize the effects of variable concentration among different samples and imported into the SIMCA software (Version 15.0, Sartorius Stedim Biotech, Umea, Sweden). The variables (spectral data) were mean Pareto scaled. Pareto scaling, i.e. each variable is divided by the square root of the standard deviation, gives greater weight to the NMR data variables with less intensity but is not as extreme as using unscaled data^76^.

Different procedures for multivariate statistical analyses of NMR data were used: Principal component analysis (PCA) and orthogonal partial least squares discriminant analysis (OPLS-DA).

A PCA was performed in the spectral data set to evaluate the homogeneity of the samples (controls and IEI-EMF) and identify any possible trends and/or outliers between the samples^77^. OPLS-DA was used to reduce model complexity and to better highlight samples discrimination. OPLS-DA a supervised classification technique and maximizes the covariance between the measured data of the X-variable (peak intensities in NMR spectra) and the response of the Y-variable (class assignment) within the groups. The goodness of the model was evaluated using a sevenfold cross-validation and “permutation test” (500 times). The permutation test was calculated by randomizing the Y-matrix (class assignment or continuous variables) while the X-matrix (peak intensity in NMR spectra) was kept constant. The permutation plot then displays the correlation coefficient between the original y-variable and the permuted y-variable on the x-axis versus the cumulative R2 and Q2 on the y-axis and draws the regression line. The intercept is a measure of the overfit, Q2Y intercept value less than 0.05 is indicative of a valid model. The estimated predictive power of the models was expressed by R2Y and Q2Y, which represent the fraction of the variation of Y-variable and the predicted fraction of the variation of Y-variable, respectively. A good prediction model is achieved when Q2 > 0.5. To highlight potential metabolites that mainly contributed to group separation, an S-line plot for the OPLS-DA model was created. The S-line is a customized S-plot for NMR spectroscopy data and combines the *covariance* (peak height) and *correlation* (color code) for the model variables displaying both in a single graph. In particular, red signals in the spectra corresponded to metabolites with greater contribution to the separation between the groups than blue signals, while the observed phase of the resonance signals on the predictive component reflects the decrease or increase (negative or positive peaks) of metabolite level in the groups. The *p(ctr)* is the centered loading vector of the first principal component.

### Univariate statistical analysis for ^1^H-NMR data

The statistical significance of the differences in metabolite concentrations, quantified by using Chenomx NMR suite 7.1, was calculated by using using the Mann–Whitney U test and a p-value < 0.05 was considered statistically significant. The Holm-Bonferroni adjustment was subsequently applied to the obtained p-values to acquire the level of significance for multiple testing^78^.

### Pathways analysis

The identified metabolites and their average relative intensities were analyzed using the pathway topology search tool in MetaboAnalyst program^79^. The global test and relative-betweenness centrality were selected for pathway enrichment analysis and the pathway topology analysis, respectively. The relative-betweenness centrality estimates the number of shortest paths going through the node.

## Supplementary information

Supplementary Information.
