# Supplementary material for: Metabolomics and psychological features in fibromyalgia and electromagnetic sensitivity
Source: Sci Rep. 2020 Nov 24;10:20418. doi: 10.1038/s41598-020-76876-8 (PMC7686375; doi:10.1038/s41598-020-76876-8)
Supplement: Supplementary file 1 — Supplementary Information. [file 41598_2020_76876_MOESM1_ESM.doc]

**Metabolomics and psychological features in fibromyalgia and electromagnetic sensitivity**

Cristina Piras^1*^, Stella Conte^2^, Monica Pibiri^1^, Giacomo Rao^3^, Sandro Muntoni^1^, Vera Piera Leoni^1^, Gabriele Finco^4^ and Luigi Atzori^1^

^1^ Department of Biomedical Science, University of Cagliari, Cagliari, Italy

^2^Department of Education, Psychology and Philosophy, University of Cagliari, Cagliari, Italy

^3^National Institute for Occupational Accident Insurance (INAIL), Rome, Italy.

^4^Department of Medical Sciences, University of Cagliari, Cagliari, Italy

***Corresponding Author:** Cristina Piras PhD**;** Department of Biomedical Sciences; Clinical Metabolomics Unit; University of Cagliari; Blocco A, Cittadella Universitaria, Monserrato (CA); Italy; Phone: +39 0706758394; email: [cristina.piras@unica.it](mailto:cristina.piras@unica.it)

**MATERIALs AND METHODs**

|  | | |
| --- | --- | --- |
| ***Chemical Questionnaire*** |  |  |
|  | **Yes** | **No** |
| Do you live in big Town? |  |  |
| Do you live in the country site? |  |  |
| Do you live in little Town? |  |  |
| Do you live near a main road? |  |  |
| Are there a gas stove or a gas heating in your home? |  |  |
| Do you smoke? |  |  |
| Are there any smokers in your family? |  |  |
| Are there coal fires near your home? |  |  |
| Are there industries of basic Chemicals near your home? |  |  |
| Are there industries of pesticides and other agrochemical products near your home? |  |  |
| Are there industries of paints, varnishes and similar coatings, printing ink and mastics near your home? |  |  |
| Are there industries of pharmaceuticals, of detergents, perfumes and toiletries manufacture of synthetic fibres near your home? |  |  |
| Are there steel industries near your home? (less than 5 kilometers) |  |  |
| Are there refineries near your home? (less than 5 kilometers) |  |  |
| Are there Dumps of waste disposal near your home? (less than 5 kilometers) |  |  |
| Are there tanneries near your home? |  |  |
| Is there the airport near your home? (less than 10 kilometers) |  |  |
| Are there coal fires near your worksite? |  |  |
| Are there industries of basic Chemicals near your worksite? |  |  |
| Are there industries of pesticides and other agrochemical products near your worksite? |  |  |
| Are there industries of paints, varnishes and similar coatings, printing ink and mastics near your worksite? |  |  |
| Are there industries of pharmaceuticals, of detergents, perfumes and toiletries manufacture of synthetic fibres near your worksite? |  |  |
| Are there steel industries near your worksite? (less than 5 kilometers) |  |  |
| Are there refineries near your worksite? (less than 5 kilometers) |  |  |
| Are there Dumps of waste disposal near your worksite? (less than 5 kilometers) |  |  |
| Are there tanneries near your worksite? |  |  |
| Is there the airport near your worksite? (less than 10 kilometers) |  |  |
| Have you ever lived near a nuclear power plant (less than 20 km) |  |  |

***Electromagnetic Questionnaire***

**1)** Do you use a mobile phone at home or at work?

How long have you been using it?

How much do you use it to make calls per day? (hours/minutes)

Have you noticed any relation to your health problems? _____________________

**2)** Do you have a cordless phone (DECT base station) at home or at work? _____

How long have you had it? Months/years.

How much do you use it to make calls per day?

Have you noticed any relation to your health problems?

**3)** Do you use wireless internet access? (Wi Fi, WLAN, WiMax, UMTS) at home or at
work?

How long have you been using it? Months/years.

How much do you use it per day? Hours/minutes

Have you noticed any relation to your health problems?

**4)** Do you use energy efficient light bulbs in your immediate vicinity? (desk lamp,
dining table lamp, reading lamp, bedside lamp) at home or at work? ______________

If yes, how long have you been using it? Months/years.

How much do you use it per day? Hours/minutes

Have you noticed any relation to your health problems?

**5)** Is there a mobile tower near your home or your workplace? (specify)

If yes, how long has it been there? Months/years. __________________________
At what distance is it from your home?

Have you-noticed any relation to your health problems? _____________________________

**6)** Are there any power lines, transformer stations or railway lines near your home or workplace? _

**8)** Do you use at home electrical apparatus (i.e. refrigerator, microwave, TV, WI-FI?)

If yes with which one? _________________________________________________________

Did you note if the health problems increase? _____________

**9)** Is there a place where the health problems increase? _____________________________

**10)** Is there a place where the health problems increase or are particularly severe? ______

***Evaluation psychological characteristics***

***Big Five Questionnaire***

In the Italian version of the Big Five Questionnaire [BFQ] [1], the main dimensions are:

*Energy*: this term is associated with the word “Extraversion” [2]. This dimension is organized into two sub-dimensions: “Dynamism”, referring to expansiveness and enthusiasm, and “Dominance”, referring to assertiveness and confidence [1].

*Friendliness*: this term is associated with the word “Agreeableness” [2]. This dimension is organized into two sub-dimensions: “Cooperativeness/Empathy”, referring to sensitiveness towards the other’s needs, and “Politeness”, referring to kindness and trust [1].

*Conscientiousness*: this term is related to the impulse control in both its proactive and inhibitory aspects ^4^. This dimension is organized into two sub-dimensions: “Scrupulousness”, referring to orderliness and precision and “Perseverance”, referring to capability of fulfilling one’s own tasks and commitments.

*Emotional Stability*: this term refers to the aspects of “negative affectivity” and “Neuroticism” [2]. This dimension lies onto two sub-dimensions: “Emotion Control”, referring to cope with one’s own emotionality and “Impulse Control”, referring to control irritation and anger.

*Openness*: this term refers to the factor “Openness to Experience” [2]. This big dimension lies onto two sub-dimensions: “Openness to Culture”, referring to the moodiness of one’s own cultural interest, and “Openness to Experience”, referring to the interest towards different cultures, people, habits, and style of life.

Lie is a “control factor” and it is meant to assess the “social desirability” type of the response set.

The BFQ is made up of 132 sentences equally distributed into 11 scales.

For these factors, raw scores have been converted into standard scores (T scores) with mean 50 and standard deviation 10.

***Stai-Y***

The State-Trait Anxiety Inventory (STAI) is a psychological inventory based on a 4-point Likert scale and consists of 40 questions on a self-report basis. The STAI measures two types of anxiety: **1)** state anxiety and **2)** trait anxiety. Higher scores in the test are positively correlated with higher levels of anxiety.

Spielberg et al. [3] developed this test with the goal of creating a set of questions allowing to assess different types of anxiety. This test was a breakthrough in this research area for all previous questionnaires focused on one type of anxiety only.

In fact, the State Anxiety Scale evaluates the current state of anxiety, asking how respondents feel “right now,” using items that measure subjective feelings of apprehension, tension, nervousness, worry, and activation/arousal of the autonomic nervous system. The Trait Anxiety Scale evaluates relatively stable aspects of “anxiety proneness,” including general states of calmness, confidence, and security. The STAI is made up of 20 items allocated to each sub-Scale for a total of 40 items. Short versions responses for the State Anxiety scale assess the intensity of current feelings “at this moment”: 1) not at all; 2) somewhat; 3) moderately so; 4) very much so.

Responses for the Trait Anxiety scale assess the frequency of feelings “in general”: 1) almost never; 2) sometimes; 3) often; 4) almost always.

The STAI-Y has been extensively used in a number of chronic medical conditions, including rheumatic diseases such as rheumatoid arthritis, systemic lupus, erythematosus, FM, and other musculoskeletal disorders [4, 5].

The range of scores for each subtest is 20–80, the highest score indicating greater anxiety. A cut point of 48-50 has been suggested to detect clinically significant symptoms for the Bith scales.

***Locus of Control***

Locus of control affects an individual’s future expectations and subjective well-being, thereby determining an individual’s psychological health [6]. Locus of control refers to people’s premise of controlling their lives. People feeling themselves responsible for what they do, have an internal locus of control. In contrast, people feeling outside events responsible for what they live, have an external locus of control [6].

Locus of control affects both physiological and psychological health and could cause psychological problems and hopelessness [6]. Furthermore, Erol et al. [7] proposed that internal locus of control is negatively related to depression and general stress. Internal locus of control could be fundamental for the ability to shift one’s perspective in everyday behavior in a positive sense [8].

In this current study, we had inserted the levels of the agreement at the items from the Locus of Control Test. At the subjects were shown the Locus Test statements regarding their beliefs [9] and they were required to agree with them as follows: agree, neither in agreement nor in disagreement, enough to disagree, completely disagree. In our version, a higher score corresponds to a more internal locus of control.

**RESULTS**

***Metabolomics analysis***


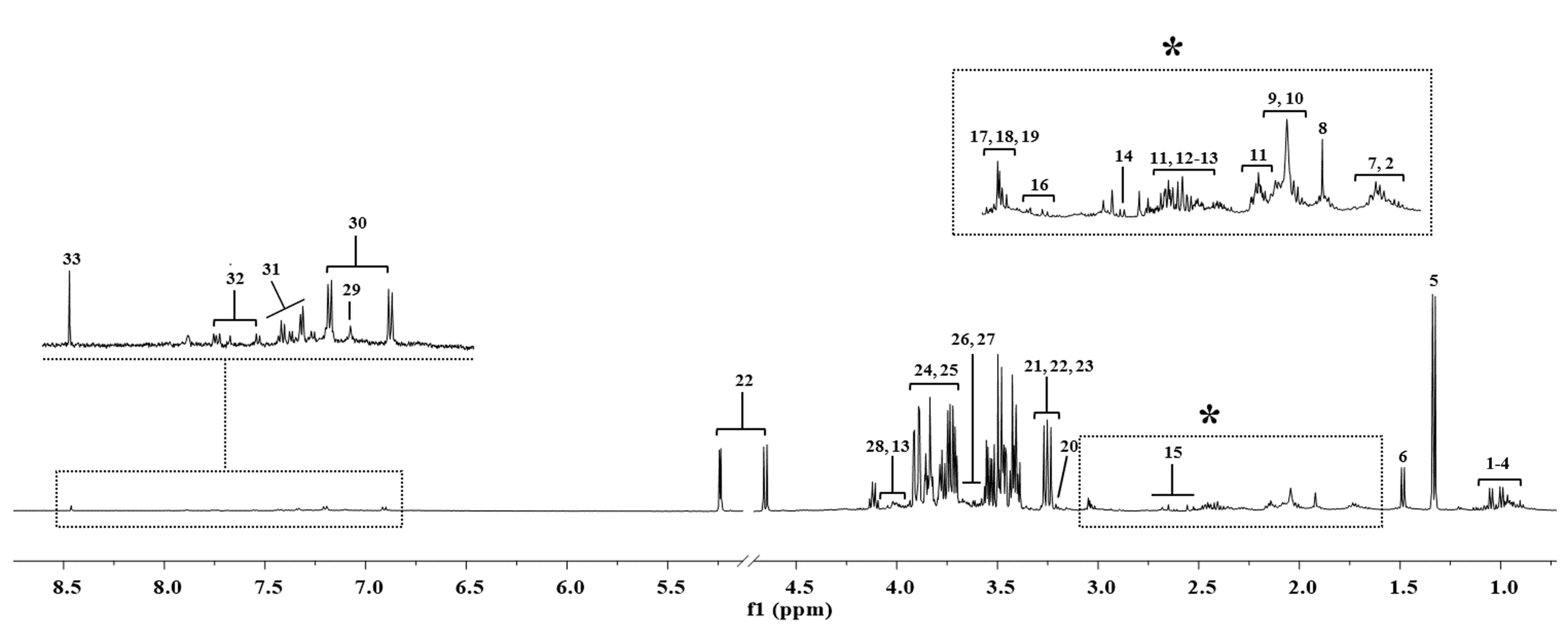


**Figure S1.** Spectral area assignments of a representative ^1^H-NMR spectra of plasma. Metabolites were identified on the basis of literature information and by using a dedicated library, such as the Human Metabolome Database (HMDB, http://www.hmdb.ca) and the 500 MHz library from Chenomx NMR suite 7.1.

Peaks: **1**, Isoleucine; **2**, Leucine; **3**, Valine, **4**, 2-Hydroxyisocaproate; **5**, Lactate; **6**, Alanine; **7**, Lysine; **8**, Acetate; **9**, N-acetyl groups; **10**, Proline; **11**, Glutamine; **12**, Glutamate; **13**, Pyroglutamate; **14**, Methylamine; **15**, Citrate; **16**, Asparagine; **17**, Creatine; **18**, Creatinine; **19**, Ornithine; **20**, Choline; **21**, sn-3-glycerophosphocoline; **22**, Glucose; **23**, Taurine; **24**, Guanidoacetate; **25**, Glycine; **26**, Myo-Inositol; **27**, Glycerol; **28**, Serine; **29**, Histamine; **30**, Tyrosine; **31**, Phenylalanine; **32**, Tryptophan; **33**, Formate.


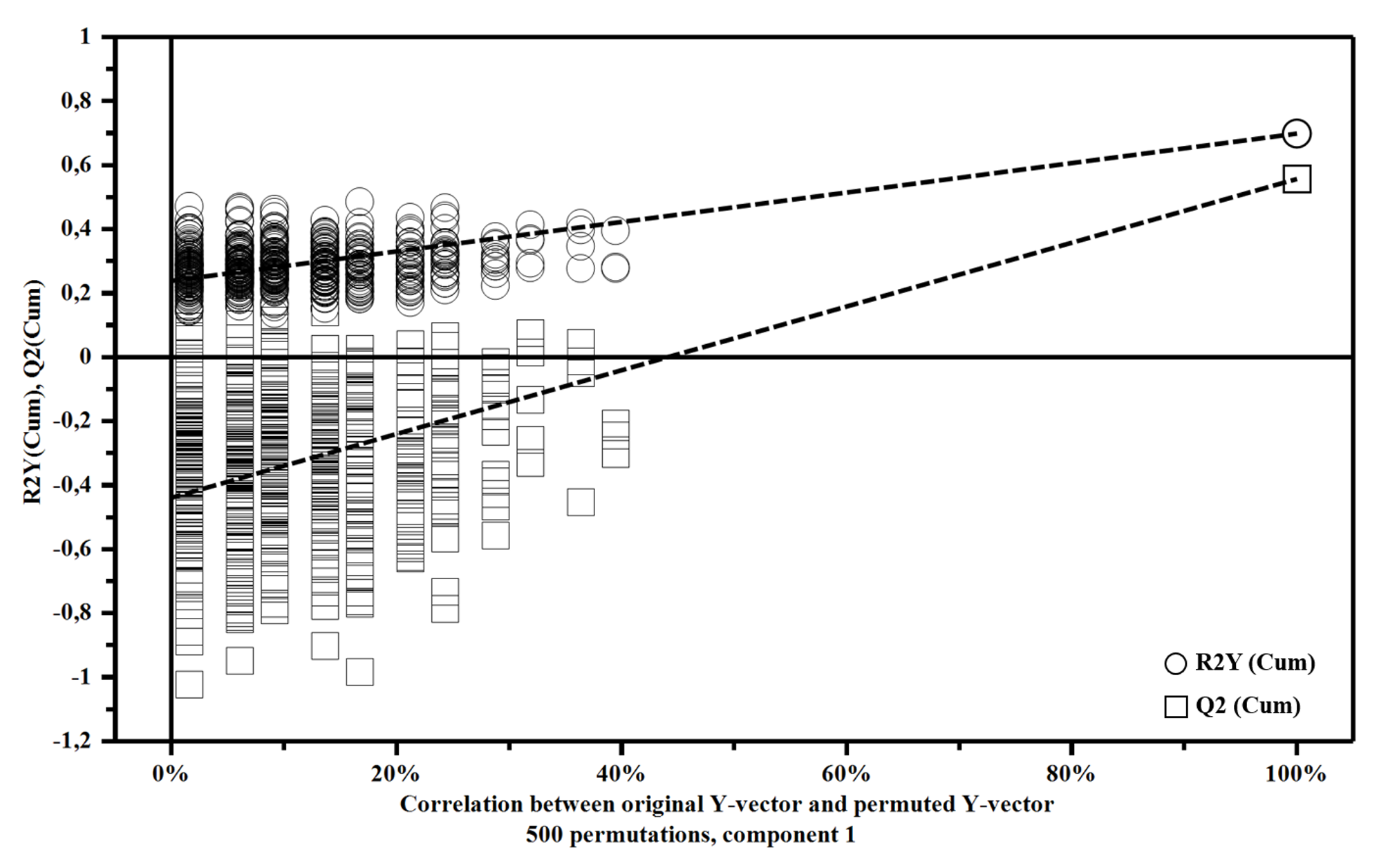


**Figure S2.** Validation plots of OPLS-DA model using a permutation test. The horizontal axis shows the correlation between the permuted and actual data, while the vertical axis displays the cumulative values of R_2_ and Q_2_. The intercept gives an estimate of the overfitting phenomenon.


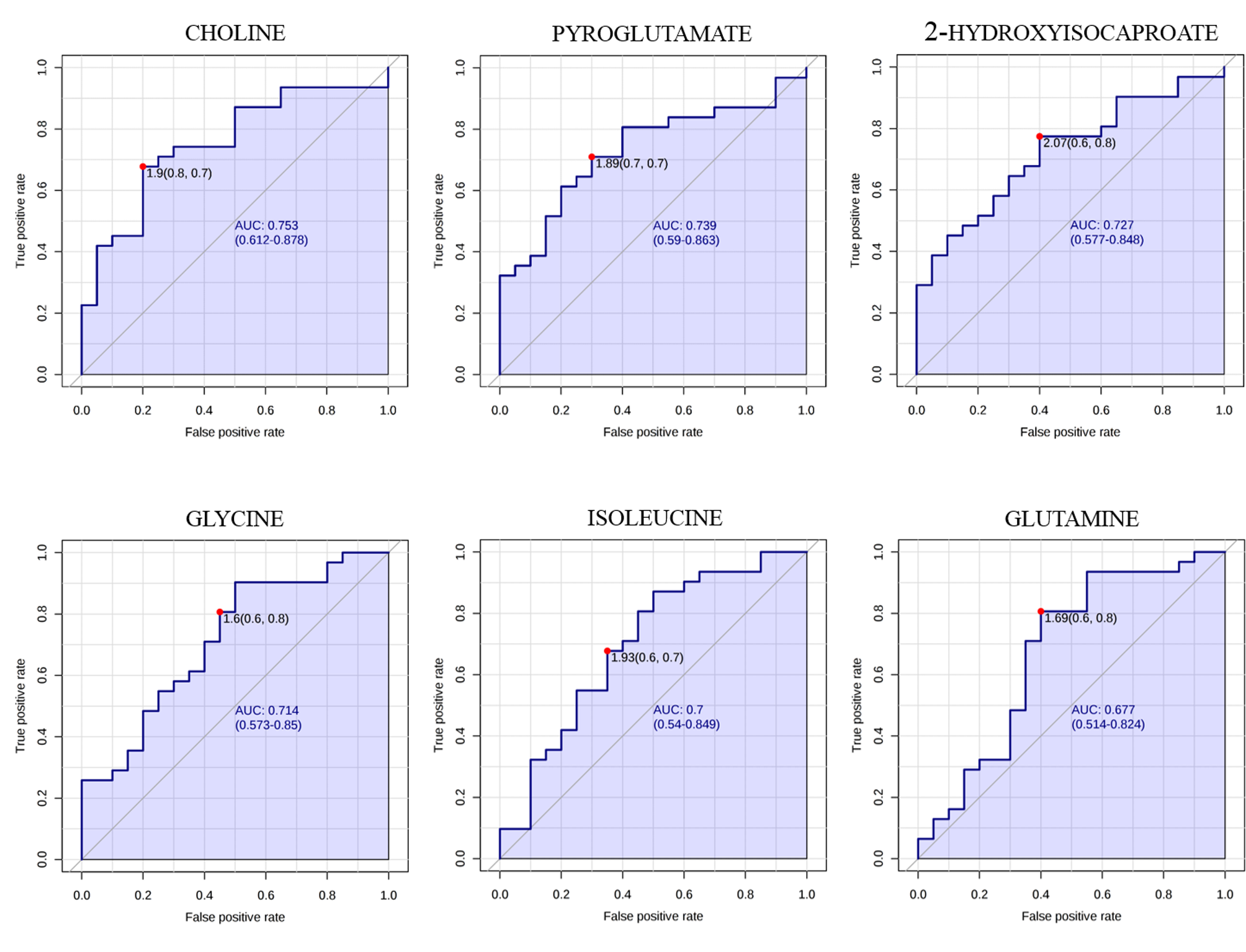


**Figure S3.** Representative ROC curves built for each altered metabolites between IEI-EMF subjects and Controls. AUC = area under the curve.


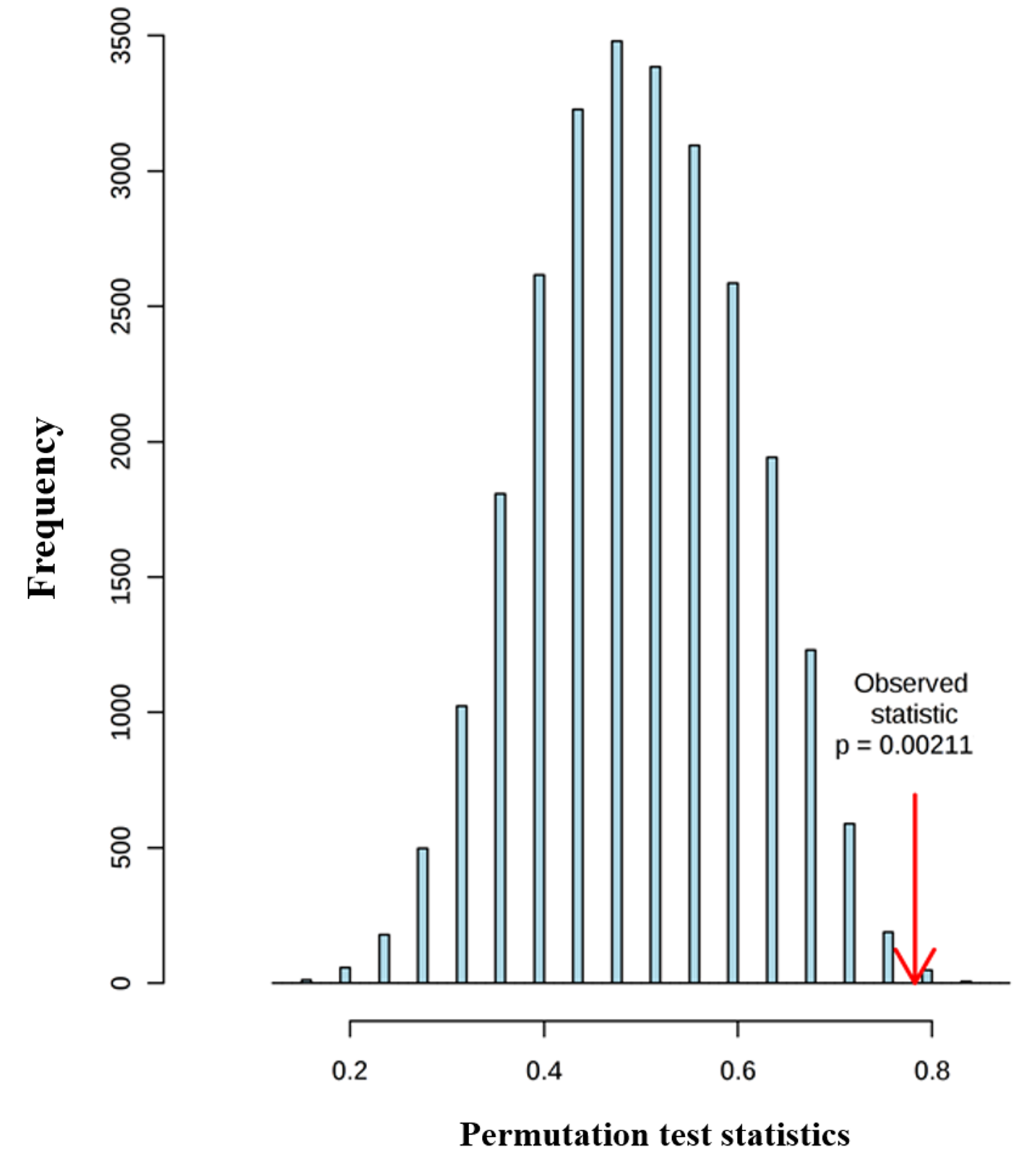


**Figure S4.** Permutation analysis (1000 times) of the predictive model.


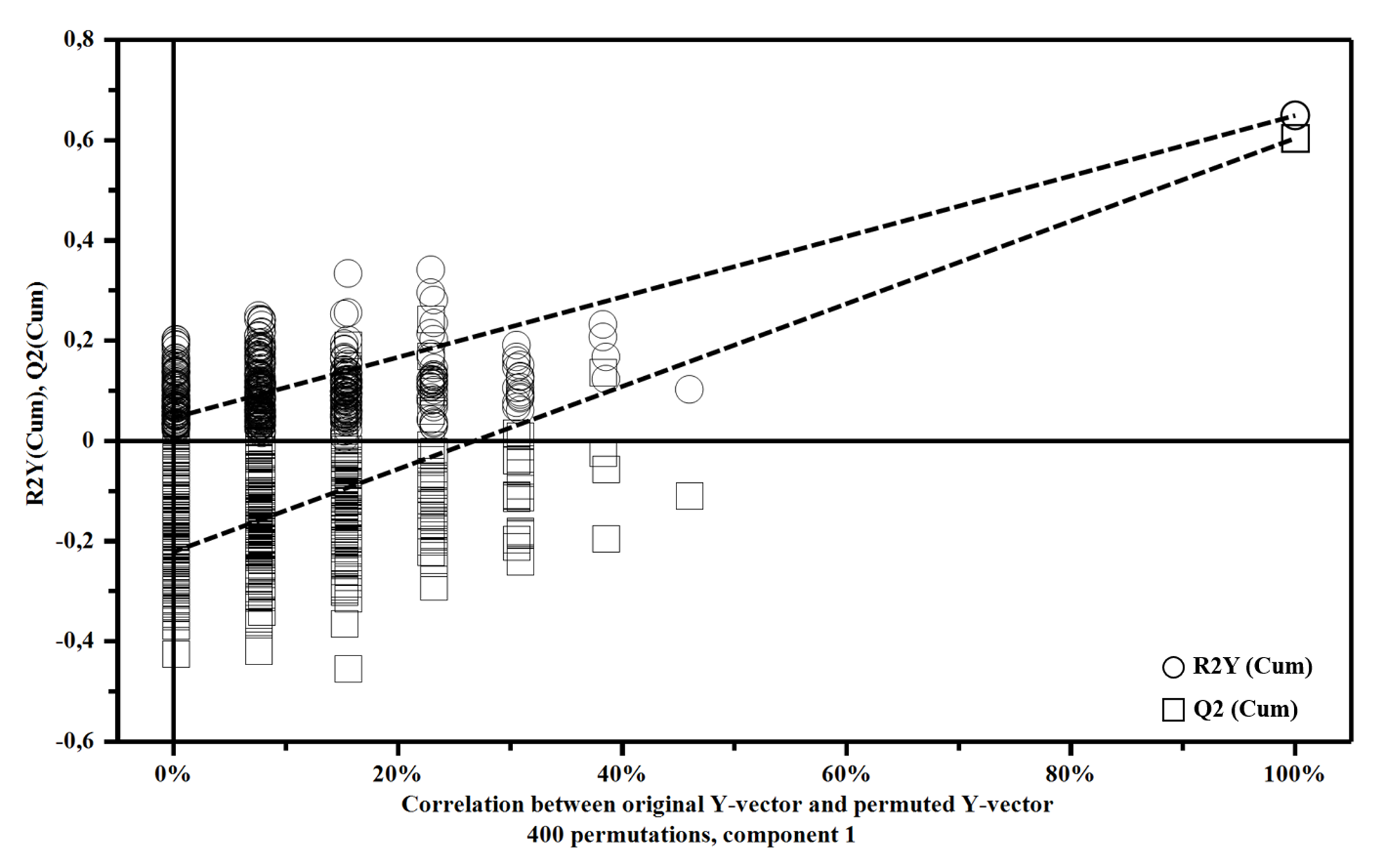


**Figure S5.** Validation plots of OPLS-DA model generated with only the identified significant metabolites using a permutation test. The horizontal axis shows the correlation between the permuted and actual data, while the vertical axis displays the cumulative values of R_2_ and Q_2_. The intercept gives an estimate of the overfitting phenomenon.


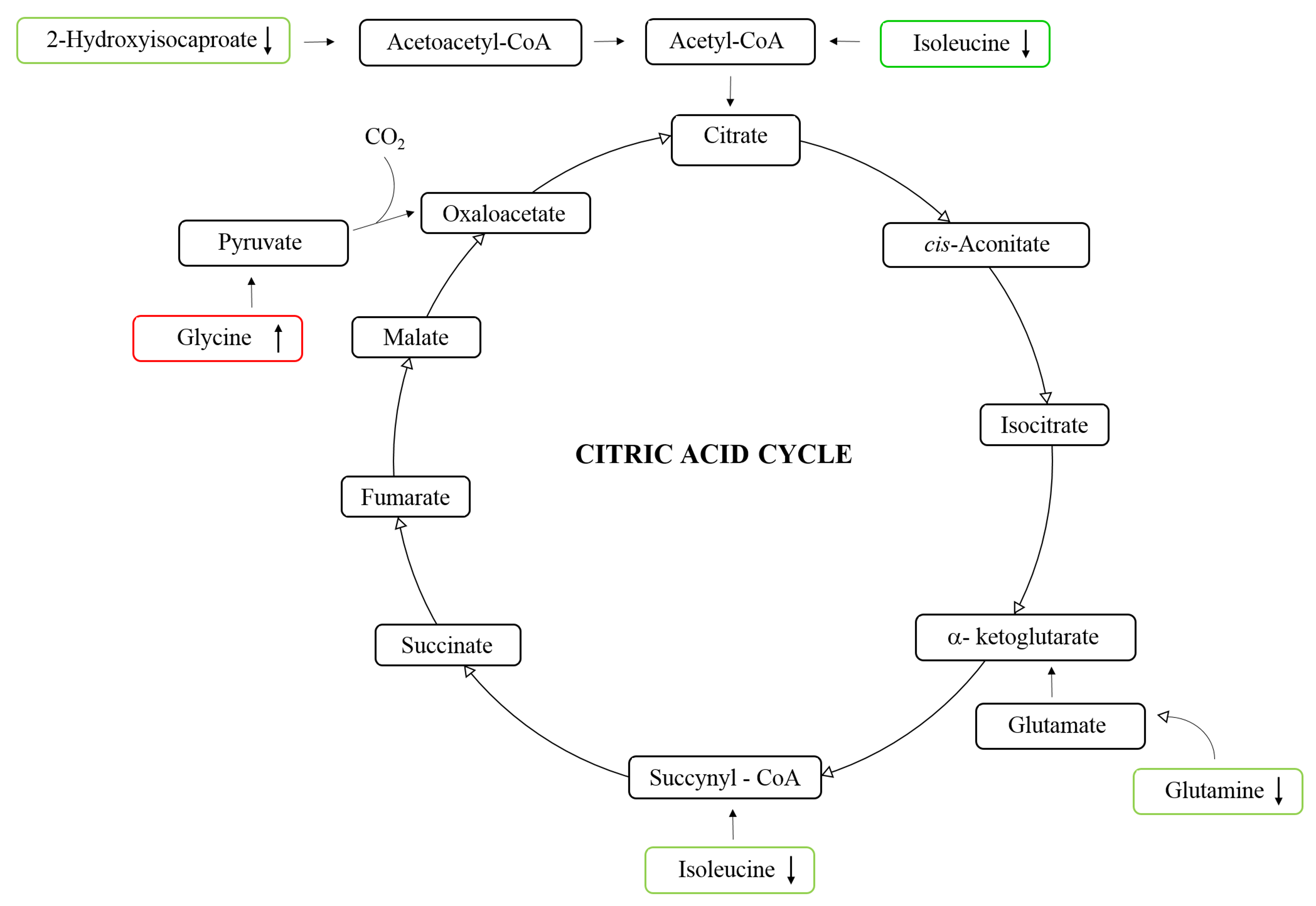


**Figure S6.** Changes in the Tricarboxylic Acid cycle pathway of IEI-EMF patients. Red color: increased concentration; green color: decreased concentration.

**Table S1**

**a)** Summary of each metabolites used to produce the linear regression model and **b)** performance values of the logistic regression models following 100-Fold Cross Validations.

| **a)** | | | | | |
| --- | --- | --- | --- | --- | --- |
|  | **Estimate** | **Std. error** | **z value** | **Pr [>|z|]** | **Odds** |
| Intercept | 0.67 | 2.706 | 0.248 | 0.804 | - |
| Choline | -1.841 | 1.313 | -1.402 | 0.161 | 0.16 |
| Pyroglutamate | 4.729 | 2.033 | 2.326 | 0.02 | 113.15 |
| Glycine | 4.408 | 2.117 | 2.082 | 0.037 | 82.14 |
| Isoleucine | -6.157 | 2.835 | -2.172 | 0.03 | 0 |
| Glutamine | -0.5 | 1.058 | -0.472 | 0.637 | 0.61 |
| 2-Hydroxyisocaproate | -0.096 | 0.709 | -0.135 | 0.893 | 0.91 |
|  |  |  |  |  |  |
| **b)** |  |  | |  | |
|  | **AUC** | **Sensitivity** | | **Specificity** | |
| Training/Discovery | 0.980  (0.970 ~ 0.989) | 0.832 (0.788 ~ 0.875) | | 0.989 (0.974 ~ 1.000) | |
| 10-fold Cross-Validation | 0.906  (0.817 ~ 0.996) | 0.871 (0.871 ~ 0.989) | | 0.900 (0.769 ~ 1.000) | |

Logistic Regression Model: logit (P) = ln (P/ (1 – P)] = α + βX where α is the intercept term, β is the regression coefficient estimated from the sample dataset, Xi; is the set of covariate (concentration) values, and the P = Pr(y=1|x) is the probability of the outcome of interest (e.g. disease case) [10].

**REFERENCES**

1. Caprara, G. V., Barbaranelli, C. & Livi, S. Mapping personality dimensions in the Big Five model. *Eur. Rev. Appl. Psychol*. **44**, 9–15 (1994).

2. McCrae, R. R. & Costa, P. T. Validation of the five-factor model of personality across instruments and observers. *J. Pers. Soc. Psychol*. **52**, 81–90 (1987).

3. Spielberger, C. D. State‐Trait Anxiety Inventory. *The Corsini Encyclopedia of Psychology*. PaloAlto, CA: Consulting Psychologists Press (2010).

4. White, K. P., Nielson, W. R., Harth, M., Ostbye, T. & Speechley, M. Chronic widespread musculoskeletal pain with or without fibromyalgia: psychological distress in a representative community adult sample. *J. Rheumatol*. **29,** 588–94 (2002).

5. Julian, L. J. Measures of anxiety: State-Trait Anxiety Inventory (STAI), Beck Anxiety Inventory (BAI), and Hospital Anxiety and Depression Scale-Anxiety (HADS-A). *Arthritis Care Res*. **63**, S467-472 (2011).

6. Mutlu, T., Balbag, Z. & Cemrek, F. The role of self-esteem, locus of control and big five personality traits in predicting hopelessness. *Procedia - Social Behav. Sci.* **9**, 788–92 (2010).

7. Erol, R. Y. Social Support (Perceived-Received) As the Moderator Between the Relationship of Stress and Health Outcomes: İmportance of Locus of Control. <http://etd.lib.metu.edu.tr/upload/12609832/index.pdf> (2008).

8. Rotter, J. B. Generalized expectancies for internal versus external control of reinforcement. *Psychol. Monogr.* **80**, 1–28 (1996).

9. Seligman, M. E. P. Authentic happiness: Using the new positive psychology to realize your potential for lasting fulfillment. New York: Free Press 336 (2002).

10. Xia, J., Sinelnikov, I. V., Han, B. & Wishart, D. S. MetaboAnalyst 3.0-making metabolomics more meaningful. *Nucleic Acids Res*. **43**, W251–W257 (2015).
